# Supplementary material for: Maternal Inflammatory Biomarkers during Pregnancy and Early Life Neurodevelopment in Offspring: Results from the VDAART Study
Source: Int J Mol Sci. 2022 Dec 3;23(23):15249. doi: 10.3390/ijms232315249 (PMC9739845; doi:10.3390/ijms232315249)
Supplement: Supplementary file 1 [file ijms-23-15249-s001.zip › ijms-2036753-supplementary.pdf]

Title: Maternal Inflammatory Biomarkers During Pregnancy and Early Life Neurodevelopment in Offspring: Results from the VDAART Study

Supplementary Table S1: Characteristics of the inflammatory biomarkers subpopulation compared to the full VDAART population

Variable			Inflammatory Biomarker Subpopulation (n=491 mother-offspring pairs)		Total VDAART population (mothers n=881; Offspring n=816)		p-value
			mean/n	SD/%	mean/n	SD/%	
Maternal Characteristics	Age at recruitment	(yrs)	27.7	5.4	27.4	5.6	0.489
	Race/Ethnicity	Black - Hispanic	21	4.30%	43	4.90%	0.968
		Black - Non-Hispanic	187	38.10%	341	38.70%	
		Other- Hispanic	42	8.60%	81	9.20%	
		Other - Non-Hispanic	38	7.70%	65	73.80%	
		White- Hispanic	65	13.20%	120	13.60%	
		White - Non-Hispanic	138	28.10%	231	26.20%	
	Study Site	Boston	143	29.10%	262	29.70%	0.971
		San Diego	169	34.40%	300	34.10%	
		St Louis	179	36.50%	319	36.20%	
	Education	Less than college grad	307	62.50%	590	67.00%	0.110
		College grad or higher	184	37.50%	291	33.00%	
	Marital Status	Not living with Father	126	25.70%	245	27.80%	0.427
		Married/Cohabiting	365	74.30%	636	72.20%	
	Number previous pregnancies at enrollment	0	173	35.23%	305	34.62%	0.989
		1	125	25.46%	230	26.11%	
		2	91	18.53%	174	19.75%	
		3	50	10.18%	83	9.42%	
		4	27	5.50%	46	5.22%	
		>5	25	5.09%	43	4.88%	
	Number living children at enrollment	0	227	46.23%	403	45.74%	0.888
		1	152	30.96%	272	30.87%	
		2	78	15.89%	140	15.89%	
		3	20	4.07%	41	4.65%	
		4	13	2.65%	19	2.16%	
		>5	1	0.20%	6	0.68%	
	Treatment Arm	Placebo	254	51.70%	439	49.80%	0.536
		Vitamin D	237	48.30%	442	50.20%	
Offspring Characteristics	Gestational age at delivery	(weeks)	39.2	1.59	38.9	2.27	0.007
	Gestational Age <37 weeks	Yes	34	6.90%	76	9.3%	0.159
	Sex	Female	242	49.30%	388	47.5%	0.615
		Male	249	50.70%	428	52.5%	
	Race/Ethnicity[a]	Black - Hispanic	40	8.10%	52	6.4%	0.179
		Black - Non-Hispanic	193	39.30%	303	37.1%	
		White- Hispanic	62	12.60%	142	17.4%	
		White - Non-Hispanic	104	21.20%	186	22.8%	
		Other- Hispanic	54	11.00%	81	9.9%	
		Other - Non-Hispanic	38	7.70%	53	6.5%	
	Weight at birth	(grams)	3311.2	504	3258.7	596.9	0.090
	Length at birth	(cm)	50.8	2.9	50.6	3.5	0.101
	Head circumference at birth	(cm)	34.1	1.9	34	2.1	0.262
	Exclusive breast feeding for first 4 months of life	Yes	157	32.00%	247	30.3%	0.801
		No	300	61.10%	491	60.2%	
		Missing	34	6.90%	78	9.6%	

Supplementary Table S2: Correlation between maternal inflammatory biomarkers

	**Early Pregnancy CRP**<br>**r (*p-value*)**	**Late Pregnancy CRP**<br>**r (*p-value*)**	**Early Pregnancy IL8**<br>**r (*p-value*)**	**Late Pregnancy IL8**<br>**r (*p-value*)**
**Early Pregnancy CRP**	**1.000**			
**Late Pregnancy CRP**	**0.677** ***(<2.2x10^{-16})***	**1.000**		
**Early Pregnancy IL8**	0.055 *(0.220)*	−0.045 *(0.315)*	**1.000**	
**Late Pregnancy IL8**	**0.191** ***(2.08x10^{-5})***	**0.124** ***(0.006)***	**0.160** ***(3.62x10^{-4})***	**1.000**

Pearson Correlation was used to define rho, p-values are shown in brackets

Supplementary Figure S1: Upset plot showing the timepoints for which 491 offspring had a completed Ages and Stages Questionnaire

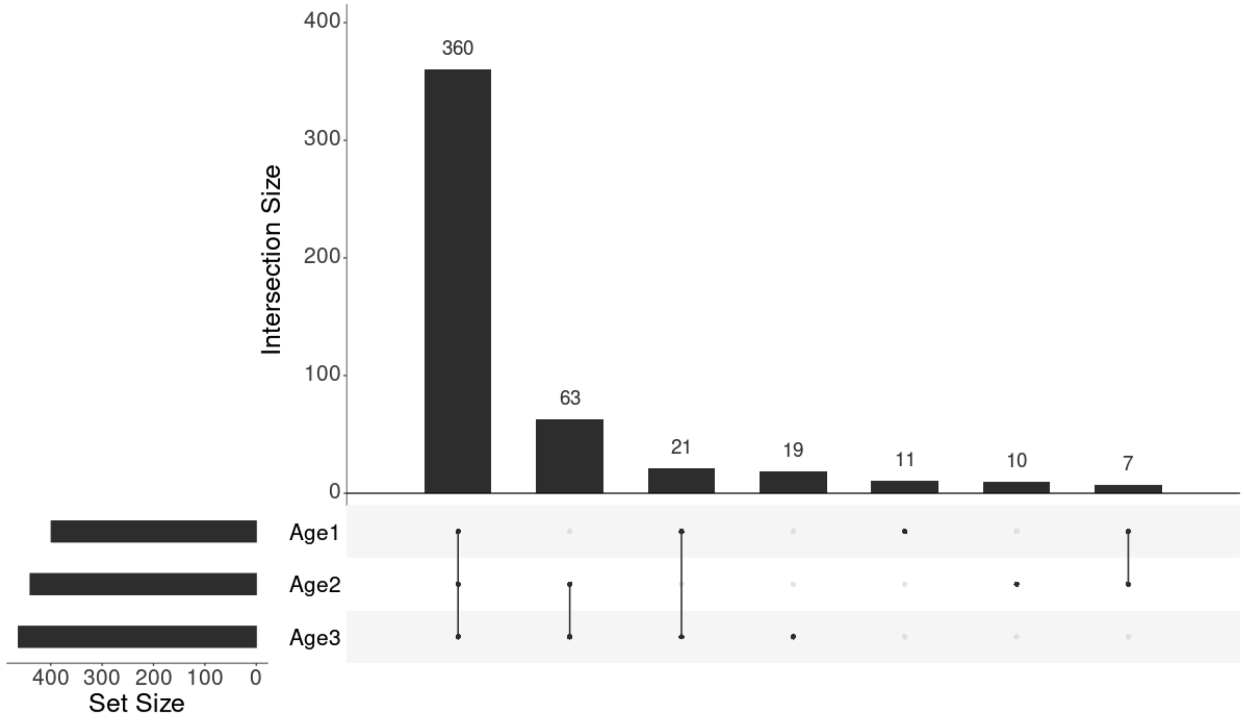

Supplementary Figure S2: Correlation between Ages and Stages Questionnaire domains across ages one, two and three

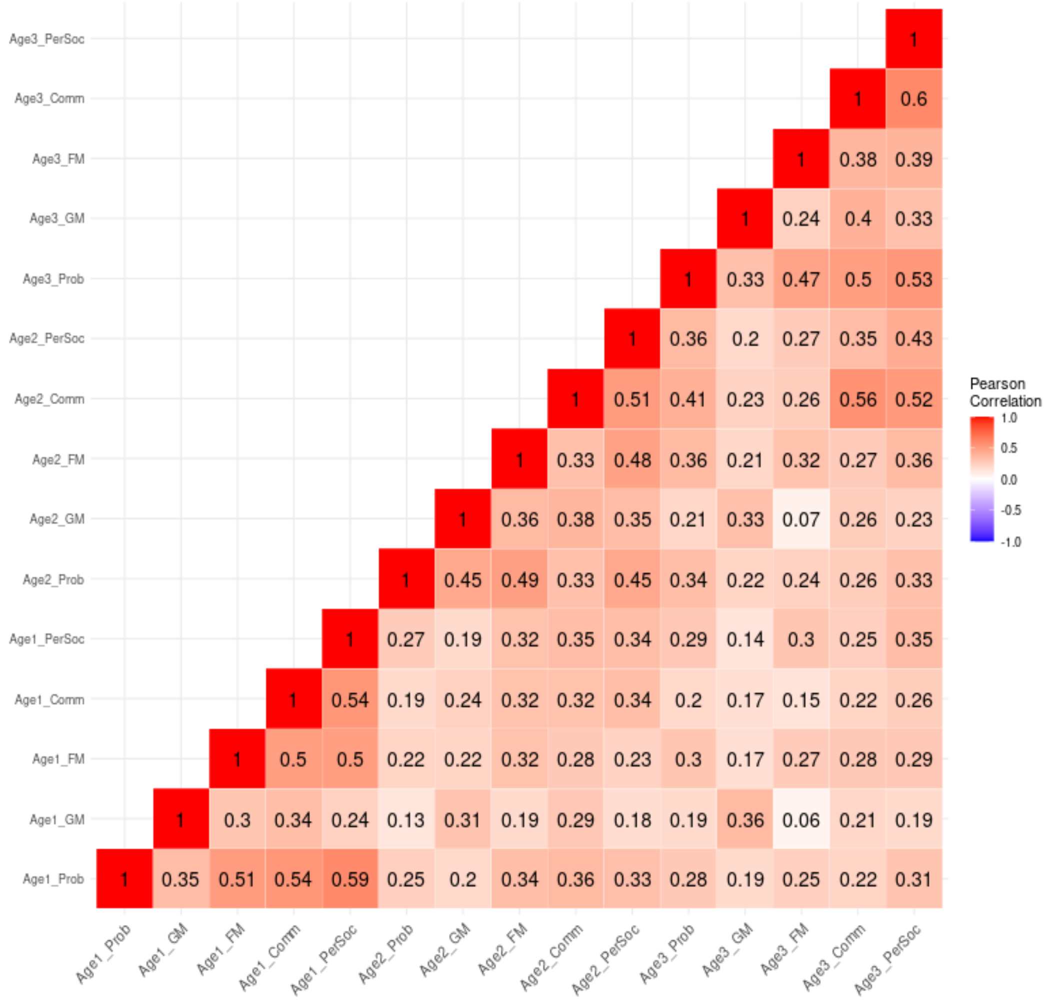

Pearson Rho is shown within the heatmap for each pairwise comparison
PerSoc; Personal-social score
Comm; Communication score
FM; Fine-Motor score
GM; Gross-Motor score
Prob; Problem-solving score

Supplementary Table S3: Summary of five Ages and Stages Questionnaire domains at ages one, two and three

Age (n children)	Domain	Mean (SD)	On Schedule		Requires Monitoring		Requires Follow-up	
			n	%	n	%	n	%
**Age One (n=399)**	**Personal Social Skills**	48.2 (12.0)	351	88.0%	29	7.3%	19	4.8%
	**Communication Skills**	51.6 (9.8)	390	97.7%	5	1.3%	4	1.0%
	**Fine Motor Skills**	54.0 (8.4)	350	87.7%	43	10.8%	6	1.5%
	**Gross Motor Skills**	52.7 (10.7)	362	90.7%	30	7.5%	7	1.8%
	**Problem Solving Skills**	48.6 (11.6)	333	83.5%	43	10.8%	23	5.8%
**Age Two (n=441)**	**Personal Social Skills**	52.0 (9.0)	369	83.9%	50	11.4%	21	4.8%
	**Communication Skills**	54.1 (10.3)	387	88.0%	33	7.5%	20	4.5%
	**Fine Motor Skills**	50.9 (7.3)	391	88.9%	25	5.7%	24	5.5%
	**Gross Motor Skills**	55.7 (7.1)	393	89.3%	33	7.5%	14	3.2%
	**Problem Solving Skills**	50.7 (10.0)	390	88.6%	40	9.1%	10	2.3%
**Age three (n=464)**	**Personal Social Skills**	54.3 (7.6)	424	91.6%	19	4.1%	20	4.3%
	**Communication Skills**	53.2 (7.9)	422	91.1%	28	6.0%	13	2.8%
	**Fine Motor Skills**	44.3 (14.7)	367	79.3%	71	15.3%	25	5.4%
	**Gross Motor Skills**	56.1 (6.8)	405	87.5%	50	10.8%	8	1.7%
	**Problem Solving Skills**	53.0 (9.8)	399	86.2%	41	8.9%	23	5.0%

Each domain consists of six age-specific questions concerning a child's ability to perform a task, with the parent responding "Yes" (10 points), "Sometimes" (5 points) or "Not yet" (0 points) which are summed to generate a domain score.
SD- Standard deviation
To determine categories, domain specific scores are compared to the expected mean score obtained from a reference distribution of scores for that age group and categorized as: (i) "On Schedule for developing normally" (above the mean); (ii) "Requires Monitoring" (1–2 standard deviations below the mean); (iii) "Needs further evaluation" (>2 standard deviations below the mean).

Supplementary Table S4: Associations between 1st and 3rd trimester levels of CRP and IL3 with skills scores in the five domains of the ASQ at ages 1,2, and 3

		**1stTrimester.CRP β (95% CI)** ***p-value***	**3rdTrimester.CRP β (95% CI)** ***p-value***	**1stTrimester.IL8 β (95% CI)** ***p-value***	**3rdTrimester.IL8 β (95% CI)** ***p-value***
**Personal-social skills score**	**Age1yrs**	-0.445 (-1.488,0.598) *0.402*	-0.142 (-1.282,0.997) *0.806*	0.052 (-0.844,0.947) *0.910*	-0.072 (-1.157,1.014) *0.897*
	**Age2yrs**	-0.206 (-0.942,0.529) *0.582*	-0.069 (-0.858,0.719) *0.863*	-0.844 (-1.452,-0.237) 0.007*	-0.272 (-0.988,0.443) *0.455*
	**Age3yrs**	-0.135 (-0.749,0.478) *0.665*	-0.346 (-1.01,0.318) *0.306*	-0.260 (-0.757,0.238) *0.305*	0.466 (-0.145,1.077) *0.134*
**Problem solving skills score**	**Age1yrs**	-1.028 (-2.022,-0.035) *0.043**	-0.914 (-2.001,0.173) *0.099*	-0.311 (-1.168,0.545) *0.475*	0.157 (-0.882,1.195) *0.767*
	**Age2yrs**	0.040 (-0.792,0.872) *0.925*	-0.127 (-1.018,0.764) *0.780*	-1.221 (-1.904,-0.538) *$4.9x10^{-4**}$*	-0.093 (-0.903,0.716) *0.821*
	**Age3yrs**	-0.083 (-0.889,0.723) *0.840*	0.009 (-0.864,0.882) *0.984*	-0.410 (-1.063,0.243) *0.218*	0.291 (-0.512,1.095) *0.477*
**Gross motor skills score**	**Age1yrs**	-0.331 (-1.263,0.601) *0.485*	-0.665 (-1.681,0.351) *0.199*	-0.902 (-1.697,-0.107) *0.026**	0.039 (-0.931,1.009) *0.937*
	**Age2yrs**	0.174 (-0.423,0.771) *0.568*	-0.073 (-0.712,0.567) *0.824*	-0.194 (-0.691,0.303) *0.444*	0.025 (-0.556,0.606) *0.932*
	**Age3yrs**	-0.022 (-0.576,0.532) *0.937*	-0.079 (-0.679,0.522) *0.797*	-0.374 (-0.822,0.075) *0.102*	0.045 (-0.508,0.598) *0.872*
**Fine motor skills score**	**Age1yrs**	-0.400 (-1.13,0.329) *0.281*	-0.151 (-0.949,0.646) *0.709*	-0.880 (-1.500,-0.259) *0.006**	-0.162 (-0.921,0.598) *0.676*
	**Age2yrs**	-0.081 (-0.698,0.535) *0.795*	-0.346 (-1.005,0.314) *0.303*	-0.919 (-1.425,-0.414) *$3.9x10^{-4**}$*	0.070 (-0.530,0.670) *0.818*
	**Age3yrs**	-0.489 (-1.659,0.68) *0.411*	-0.798 (-2.064,0.468) *0.216*	0.221 (-0.729,1.17) *0.648*	0.299 (-0.869,1.467) *0.615*
**Communication skills score**	**Age1yrs**	0.348 (-0.505,1.2) *0.423*	$-7.9x10^{-5}$ (-0.932,0.932) *0.999*	-0.564 (-1.293,0.166) *0.130*	0.058 (-0.829,0.946) *0.897*
	**Age2yrs**	-0.448 (-1.287,0.39) *0.294*	-0.431 (-1.329,0.467) *0.346*	-0.483 (-1.181,0.214) *0.174*	0.069 (-0.748,0.886) *0.868*
	**Age3yrs**	-0.640 (-1.265,-0.015) *0.045**	-0.725 (-1.401,-0.048) *0.036**	-0.498 (-1.006,0.009) *0.054*	-0.038 (-0.664,0.589) *0.906*

ASQ score and biomarker levels were both treated as continuous variables. Analyses were adjusted for study site, treatment group, maternal marital status and educational level and gestational age at delivery

**Significant at p<0.05*

***Significant after Benjamini-Hochberg FDR correction*

Supplementary Figure S3: (A-D) Distribution of IL8 and CRP in the early and late pregnancy, each mother is represented by a bar, mothers of children who performed poorly across all Ages and Stages Questionnaire domains in any given year are highlighted

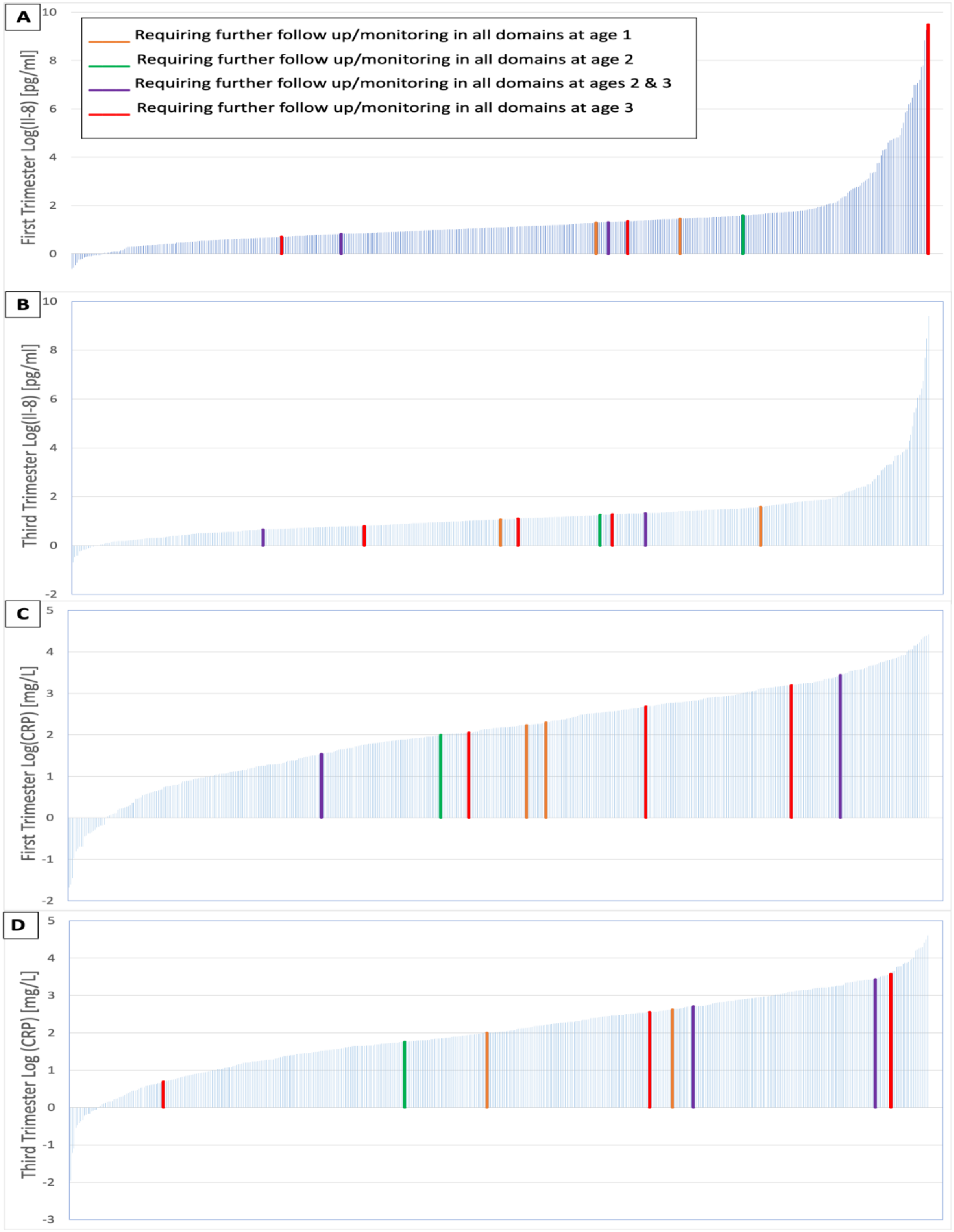

Supplementary Figure S4: Correlation between change in IL8 from the 1st to the 3rd trimester and change in CRP during the same period in 491 VDAART mothers

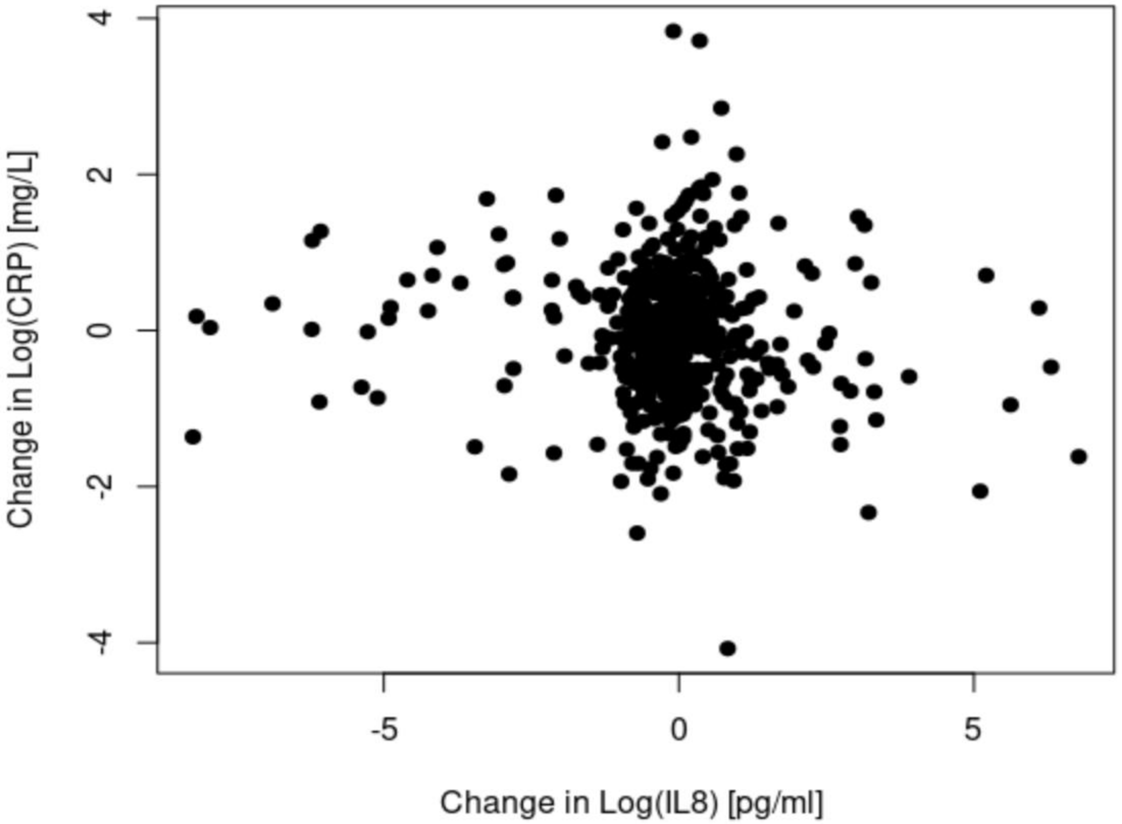

Pearson rho=−0.08, p=0.072

Supplementary Table S5: Interaction effect estimates and p-values for a maternal biomarker*child sex (Male) interaction on Ages and Stages Questionnaire domain scores at ages 1,2, and 3

		1stTrimester.CRP		3rdTrimester.CRP		1stTrimester.IL8		3rdTrimester.IL8	
		Interaction β	Interaction p-value	Interaction β	Interaction p-value	Interaction β	Interaction p-value	Interaction β	Interaction p-value
**Personal-social skills score**	**Age1yrs**	-0.723	0.494	0.279	0.809	1.437	0.109	-1.092	0.323
	**Age2yrs**	0.059	0.935	1.073	0.166	-0.391	0.513	0.610	0.393
	**Age3yrs**	0.253	0.679	0.846	0.196	-1.161	0.019*	0.333	0.586
**Problem solving skills score**	**Age1yrs**	-0.304	0.763	0.655	0.552	0.993	0.248	0.102	0.923
	**Age2yrs**	0.052	0.950	0.183	0.837	0.996	0.143	-0.079	0.923
	**Age3yrs**	0.576	0.478	0.634	0.467	-1.395	0.033*	0.332	0.684
**Gross motor skills score**	**Age1yrs**	-0.029	0.976	0.882	0.390	0.348	0.662	0.125	0.899
	**Age2yrs**	-0.895	0.136	-0.317	0.620	0.507	0.308	-0.073	0.902
	**Age3yrs**	-0.505	0.364	-0.717	0.230	-0.231	0.608	0.761	0.173
**Fine motor skills score**	**Age1yrs**	0.377	0.610	0.298	0.712	-0.340	0.585	-0.440	0.570
	**Age2yrs**	0.145	0.815	0.307	0.641	0.174	0.731	0.401	0.510
	**Age3yrs**	0.438	0.708	0.849	0.499	0.030	0.975	2.077	0.077
**Communication skills score**	**Age1yrs**	1.220	0.154	1.564	0.094	0.292	0.688	0.061	0.946
	**Age2yrs**	-0.944	0.260	0.251	0.778	0.217	0.754	0.770	0.350
	**Age3yrs**	0.363	0.562	0.132	0.844	-0.377	0.457	0.575	0.362

ASQ score and biomarker levels were both treated as continuous variables. Analyses were adjusted for study site, treatment group, maternal marital status and educational level, gestational age at delivery, child race/ethnicity, and child sex

$*p<0.05$

Supplementary Table S6: Interaction effect estimates and p-values for a maternal biomarker*breastfeeding (exclusive breast feeding for four months, Yes) interaction on Ages and Stages Questionnaire domain scores at ages 1,2, and 3

		1stTrimester.CRP		3rdTrimester.CRP		1stTrimester.IL8		3rdTrimester.IL8	
		Interaction β	Interaction p-value	Interaction β	Interaction p-value	Interaction β	Interaction p-value	Interaction β	Interaction p-value
Personal-social skills score	Age1yrs	-0.527	0.627	0.029	0.981	-0.591	0.500	-0.862	0.480
	Age2yrs	0.403	0.617	0.463	0.592	1.297	0.039*	-0.397	0.658
	Age3yrs	-0.477	0.477	0.170	0.815	-0.175	0.732	0.078	0.920
Problem solving skills score	Age1yrs	0.197	0.852	-0.946	0.417	0.324	0.706	-0.746	0.532
	Age2yrs	0.550	0.538	-0.272	0.776	1.183	0.088	0.530	0.593
	Age3yrs	0.122	0.890	1.233	0.196	-0.258	0.700	0.027	0.979
Gross motor skills score	Age1yrs	0.343	0.734	-1.336	0.228	0.056	0.944	-0.028	0.980
	Age2yrs	0.956	0.145	0.031	0.965	-0.242	0.641	-0.057	0.938
	Age3yrs	0.964	0.111	-0.198	0.762	0.276	0.548	-0.410	0.557
Fine motor skills score	Age1yrs	0.697	0.367	0.200	0.815	-0.723	0.242	-1.054	0.225
	Age2yrs	-0.055	0.934	0.003	0.997	-0.718	0.162	-0.503	0.493
	Age3yrs	-0.302	0.809	1.117	0.410	-1.574	0.098	-1.514	0.294
Communication skills score	Age1yrs	0.984	0.273	0.060	0.952	-0.534	0.461	-0.378	0.709
	Age2yrs	-0.679	0.460	-1.389	0.159	0.215	0.767	0.192	0.852
	Age3yrs	-0.394	0.563	-0.333	0.652	-0.043	0.934	-0.430	0.586

ASQ score and biomarker levels were both treated as continuous variables. Analyses were adjusted for study site, treatment group, maternal marital status and educational level, gestational age at delivery, child race/ethnicity, and child sex

**p<0.05*

Supplementary Table S7: Interaction effect estimates and p-values for a maternal biomarker*vitamin D supplementation interaction on Ages and Stages Questionnaire domain scores at ages 1,2, and 3

		1stTrimester.CRP		3rdTrimester.CRP		1stTrimester.IL8		3rdTrimester.IL8	
		Interaction β	Interaction p-value	Interaction β	Interaction p-value	Interaction β	Interaction p-value	Interaction β	Interaction p-value
Personal-social skills score	Age1yrs	0.044	0.967	-1.035	0.366	-0.091	0.919	1.153	0.300
	Age2yrs	0.514	0.487	0.809	0.305	0.090	0.883	0.622	0.396
	Age3yrs	-0.762	0.217	-1.364	0.040*	-0.422	0.395	0.039	0.950
Problem solving skills score	Age1yrs	-0.552	0.582	-0.472	0.665	0.128	0.880	-1.000	0.348
	Age2yrs	0.085	0.919	-0.250	0.779	0.350	0.611	0.420	0.613
	Age3yrs	0.635	0.434	-1.270	0.145	-0.111	0.865	-0.057	0.945
Gross motor skills score	Age1yrs	-1.115	0.235	-0.723	0.479	0.148	0.851	-0.527	0.597
	Age2yrs	-0.282	0.638	-0.009	0.988	-0.120	0.810	0.446	0.454
	Age3yrs	0.904	0.105	0.152	0.801	-0.258	0.565	0.315	0.579
Fine motor skills score	Age1yrs	-0.474	0.519	-0.757	0.345	0.523	0.396	1.164	0.135
	Age2yrs	-0.061	0.922	-0.154	0.815	-0.851	0.095	-0.332	0.590
	Age3yrs	-0.051	0.965	-0.256	0.840	0.085	0.929	1.411	0.238
Communication skills score	Age1yrs	-0.771	0.370	-1.670	0.074	-0.519	0.474	0.166	0.855
	Age2yrs	-1.285	0.127	-1.372	0.127	0.339	0.630	-1.002	0.231
	Age3yrs	0.173	0.783	-0.630	0.352	-0.201	0.691	0.943	0.141

ASQ score and biomarker levels were both treated as continuous variables. Analyses were adjusted for study site, treatment group, maternal marital status and educational level, gestational age at delivery, child race/ethnicity, and child sex
$p<0.05$

Supplementary Figure S5: Associations between 1st and 3rd trimester levels of CRP and IL8 with skills scores in the five domains of the Ages and Stages Questionnaire at ages 1,2, and 3 excluding 34 mother-child pairs with a gestational age <37 weeks

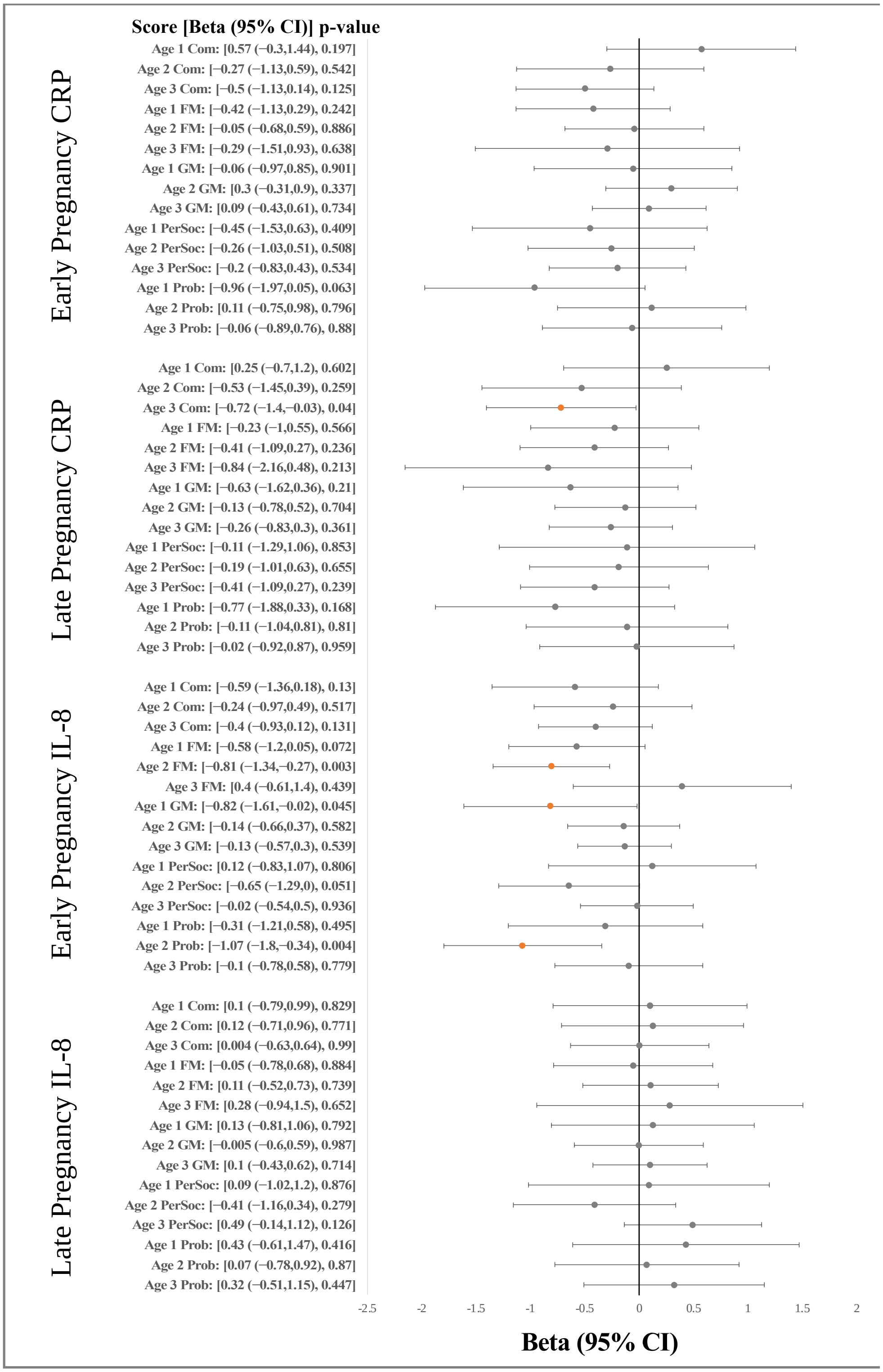

ASQ score and biomarker levels were both treated as continuous variables. Analyses were adjusted for study site, treatment group, maternal marital status and educational level and gestational age at delivery

Grey; NS

Orange: Significant at $p<0.05$

Red: Significant after Benjamini-Hochberg FDR correction

PerSoc; Personal-social score

Comm; Communication score

FM; Fine-Motor score

GM; Gross-Motor score

Prob; Problem-solving score

Supplementary Figure S6: Associations between 1st and 3rd trimester levels of CRP and IL8 with skills scores in the five domains of the Ages and Stages Questionnaire at ages 1,2, and 3 with additional adjustment for pre-pregnancy BMI

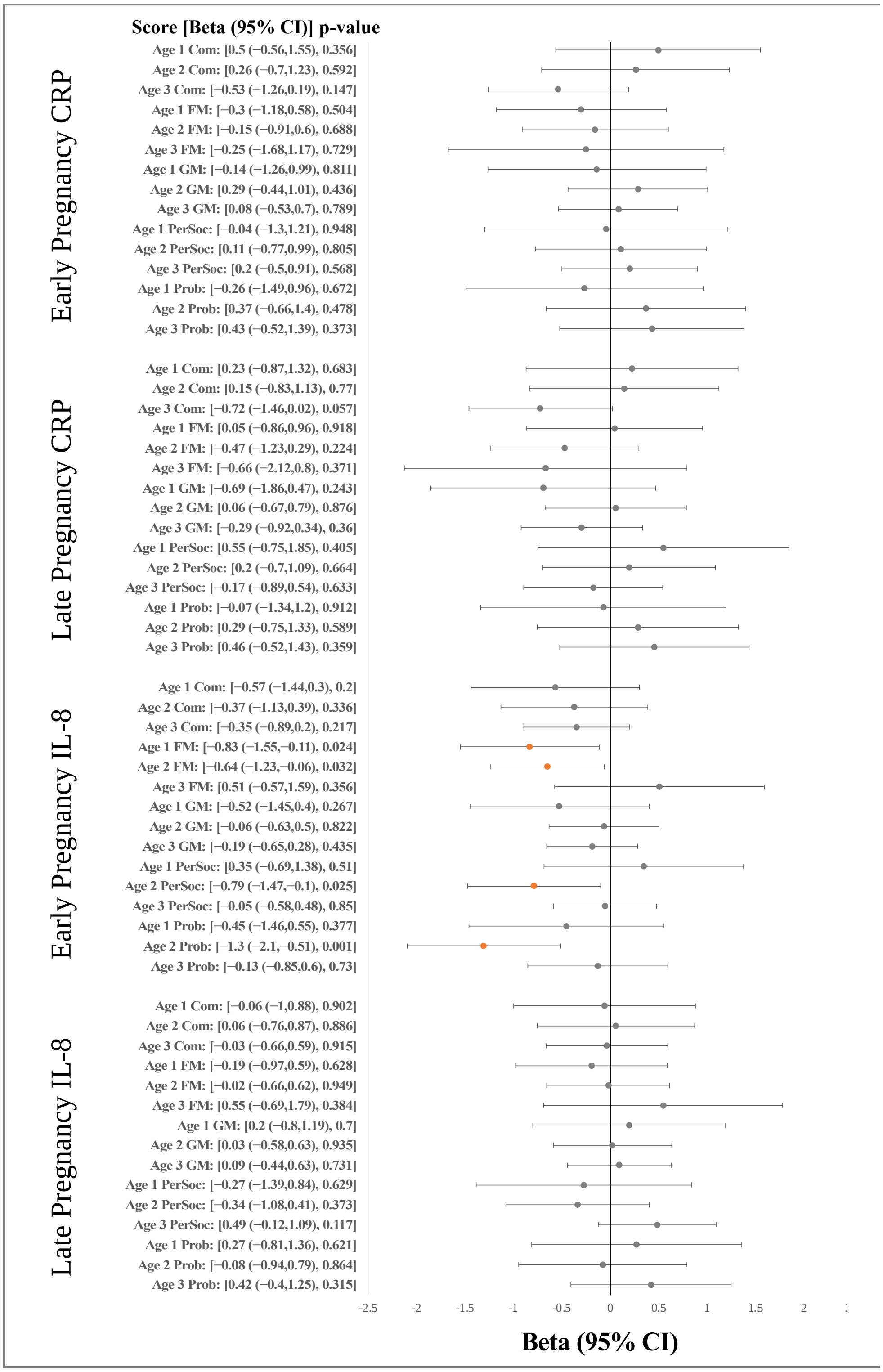

ASQ score and biomarker levels were both treated as continuous variables. Analyses were adjusted for study site, treatment group, maternal marital status and educational level, gestational age at delivery and prepregnancy BMI

Grey; NS
Orange: Significant at p<0.05
Red: Significant after Benjamini-Hochberg FDR correction
PerSoc; Personal-social score
Comm; Communication score
FM; Fine-Motor score
GM; Gross-Motor score
Prob; Problem-solving score

Supplementary Table S8: Association between prepregnancy BMI and Ages and Stages Questionnaire scores in 426 mother-child pairs

Domain	Age gp	β	Lower 95% CI	Upper 95% CI	P-value
**Personal-social skills score**	**Age1yrs**	-0.120	-0.286	0.046	0.158
	**Age2yrs**	-0.085	-0.196	0.025	0.132
	**Age3yrs**	-0.049	-0.137	0.040	0.282
**Problem solving skills score**	**Age1yrs**	-0.201	-0.363	-0.038	0.016*
	**Age2yrs**	-0.056	-0.185	0.073	0.395
	**Age3yrs**	-0.059	-0.179	0.062	0.340
**Gross motor skills score**	**Age1yrs**	-0.041	-0.191	0.108	0.587
	**Age2yrs**	-0.025	-0.116	0.066	0.592
	**Age3yrs**	0.024	-0.054	0.102	0.545
**Fine motor skills score**	**Age1yrs**	-0.055	-0.171	0.062	0.358
	**Age2yrs**	0.015	-0.080	0.110	0.755
	**Age3yrs**	-0.061	-0.241	0.118	0.503
**Communication skills score**	**Age1yrs**	0.013	-0.127	0.154	0.851
	**Age2yrs**	-0.120	-0.242	0.002	0.054
	**Age3yrs**	-0.086	-0.177	0.005	0.066

ASQ score and biomarker levels were both treated as continuous variables. Analyses were adjusted for study site, treatment group, maternal marital status and educational level, gestational age at delivery

**Significant at $p<0.05$*

Supplementary Table S9: CRP and IL-8 as non-significant mediators in the association between pre-pregnancy BMI and age one problem solving score

Independent variable	Mediator	Outcome	Direct Effect [1]	Indirect Effect[2]	Proportion Mediated[3]
Pre-pregnancy BMI	Early pregnancy CRP	Age One problem solving score	-0.183 (-0.417, 0.050)<br>*p* = 0.120	-0.022 (-0.129, 0.080)<br>*p* = 0.680	0.098 (-0.569, 1.590)<br>*p* = 0.700
Pre-pregnancy BMI	Early pregnancy IL-8	Age One problem solving score	-0.199 (-0.391, -0.020)<br>p = 0.022	-0.001 (-0.016, 0.010)<br>p=0.814	0.003 (-0.112, 0.150)<br>p=0.814
Pre-pregnancy BMI	Late pregnancy CRP	Age One problem solving score	-0.199 (-0.410, 0.010)<br>p = 0.040	-0.004 (-0.074, 0.060)<br>p=0.874	0.021 (-0.569, 1.000)<br>p=0.894
Pre-pregnancy BMI	Late pregnancy IL-8	Age One problem solving score	-0.206 (-0.405, -0.020)<br>p = 0.036	0.006 (-0.015, 0.030)<br>p=0.036	-0.022 (-0.272, 0.130)<br>p=0.604

[1]Direct effect = effect of independent variable on outcome, controlling for the mediator, reported as estimate (95% confidence interval) and associated p-value
[2]Indirect effect = average mediator effect, reported as estimate (95% confidence interval) and associated p-value
[3]Proportion mediated = proportion of effect of independent variable on outcome through the mediator (indirect effect/total effect), reported as proportion (95% confidence interval) and associated p-value
All models were adjusted for study site, treatment group, maternal marital status and educational level
